# Identifying the ‘inorganic gene’ for high-temperature piezoelectric perovskites through statistical learning

**DOI:** 10.1098/rspa.2010.0543

**Published:** 2011-03-02

**Authors:** Prasanna V. Balachandran, Scott R. Broderick, Krishna Rajan

**Affiliations:** Department of Materials Science and Engineering and Institute for Combinatorial Discovery, Iowa State University, Ames, IA 50011, USA

**Keywords:** inorganic gene, high-temperature piezoelectrics, statistical learning, information theory, data-driven modelling

## Abstract

This paper develops a statistical learning approach to identify potentially new high-temperature ferroelectric piezoelectric perovskite compounds. Unlike most computational studies on crystal chemistry, where the starting point is some form of electronic structure calculation, we use a data-driven approach to initiate our search. This is accomplished by identifying patterns of behaviour between discrete scalar descriptors associated with crystal and electronic structure and the reported Curie temperature (*T*_C_) of known compounds; extracting design rules that govern critical structure–property relationships; and discovering in a quantitative fashion the exact role of these materials descriptors. Our approach applies linear manifold methods for data dimensionality reduction to discover the dominant descriptors governing structure–property correlations (the ‘genes’) and Shannon entropy metrics coupled to recursive partitioning methods to quantitatively assess the specific combination of descriptors that govern the link between crystal chemistry and *T*_C_ (their ‘sequencing’). We use this information to develop predictive models that can suggest new structure/chemistries and/or properties. In this manner, BiTmO_3_–PbTiO_3_ and BiLuO_3_–PbTiO_3_ are predicted to have a *T*_C_ of 730^°^C and 705^°^C, respectively. A quantitative structure–property relationship model similar to those used in biology and drug discovery not only predicts our new chemistries but also validates published reports.

## 1. Introduction

Through many seminal papers, Alan McKay has expounded on the idea of a framework for ‘Generalized Crystallography’ (Mackay 1966, 1974, 1977, 1986). He has proposed that ‘the crystal is a structure, the description of which is much smaller than the structure itself’ and that this description of structure serves as a ‘carrier of information’ about the structure on larger length scales (MacKay 2002). He went on to suggest that these components of description of structure can help develop a ‘biological approach to inorganic systems’ and proposed the construction of an ‘inorganic gene’. This paradigm serves as motivation underlying the present study by exploring how fundamental pieces of information, treated as discrete bits of data, can collectively characterize the stability and properties of a given crystal chemistry. We show how the use of statistical learning tools including fundamental concepts borrowed from information theory can be used to characterize a crystal structure in terms of fundamental descriptors of information (i.e. the ‘genes’) and how these pieces of information interact or are ‘sequenced’ to guide the characteristics of that crystal structure and in fact help to guide the development of new crystal chemistries and targeted physical properties.

The challenge in defining the ‘gene’ in inorganic crystal chemistry is to characterize the appropriate combination of discrete characteristics associated with crystal chemistry that collectively define a particular property or set of properties of the material. Normally, structure–property relationships are guided by defined functional relationships (e.g. electronic structure calculations to define energy landscapes associated with crystal chemistry). However, we propose an approach to establish such a structure–property relationship where we do not assume any specific formulation linking structure with property (Jóhannesson *et al.* 2002; Curtarolo *et al.* 2003; Woodley *et al*. 2004; Dudiy & Zunger 2006; Fischer *et al.* 2006; Sluiter 2007; Mohn & Kob 2009; Oganov & Valle 2009). Rather, we take a data-driven approach where we seek to establish structure–property relationships by identifying patterns of behaviour between known discrete scalar descriptors associated with crystal and electronic structure and observed properties of the material. From this, we extract design rules that allow us to systematically identify critical structure–property relationships, resulting in identifying in a quantitative fashion the exact role of specific combination of materials descriptors (i.e. genes) that govern a given property. This is the foundation of the concept of the quantitative structure–activity (or property) relationship (QSAR/QSPR) widely used in the field of organic chemistry and drug discovery. The mathematical underpinning of developing a QSPR-type relationship is statistical learning (a term encompassing a broad range of tools derived from statistics, data mining and machine learning). In our group, we have applied this approach to explore a variety of questions associated with crystal chemistry (Suh & Rajan 2005, 2009; Gadzuric *et al.* 2006; Rajagopalan & Rajan 2007; George *et al*. 2009; Broderick *et al.* 2010; Rajan 2010, Zenasni *et al.* 2010), and in this paper, we demonstrate that by using the QSPR concept, we can identify through the tools of statistical inference, how discrete bits of information that define a robust QSPR relationship can be sequenced to help identify new materials with new and targeted properties. The specific objective of the present study is identifying, through the sole use of statistical learning methods, new high-temperature piezoelectric ferroelectrics. However, this paper also serves as a generic template for an information science-based materials discovery and design strategy, in the spirit of Mackay’s proposition of an inorganic gene.

## 2. Background

### (a) Materials chemistry of high-temperature piezoelectrics

Historically, the design of materials chemistry for high-temperature piezoelectric behaviour has been guided by an apparent linear relationship between Goldschmidt’s tolerance factor (*t*) and Curie temperature (*T*_C_) at the morphotropic phase boundary (MPB) composition of the PbTiO_3_ (PT)-based end-member solid solutions (Eitel *et al*. 2001; Duan *et al*. 2004). However, the use of the tolerance factor as a ‘figure of merit’ has had limited impact in developing or identifying new materials via experiment (Eitel *et al*. 2001; Duan *et al*. 2004) or computation (Baettig *et al*. 2005), owing to the fact that it captures only a very limited set of variables (i.e. ionic radii) describing a given perovskite crystal chemistry (Thomas 1997). The motivation of our work is to find alternative computational based methods that can help to refine the chemical search space and identify potentially new and promising piezoelectric materials for high-temperature applications.

**Figure 1.**
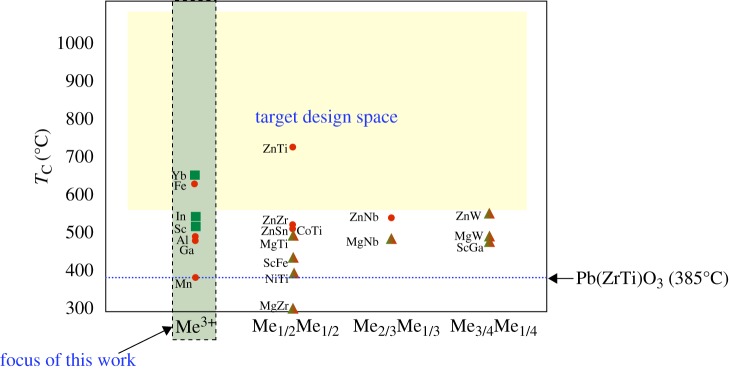
In this figure, we map the Curie temperature (*T*_C_) of known and predicted perovskite-based ferroelectric compounds in the chemical space of BiMeO_3_–PbTiO_3_ solid solution, where Me is a single cation with charge 3+ (e.g. Al, Sc, In, etc.) or a combination of two different cations Me_1/2_Me_1/2_ (e.g. ZnTi, ZnZr, ZnSn, etc.), Me_2/3_Me_1/3_ (e.g. ZnNb, MgNb) and Me_3/4_Me_1/4_ (e.g. ZnW, MgW, ScGa) with an average charge 3+ and that occupies the octahedral site of the perovskite lattice (Eitel *et al*. 2001; Grinberg *et al*. 2005; Suchomel & Davies 2005; Stein *et al*. 2006; Grinberg & Rappe 2007). The target design space represents the high-temperature regime that is of interest to us, and, as it can be clearly seen, the chemical search space is sparse in this region with as many as only three compounds being identified. For reference, *T*_C_ of PbZrO_3_–PbTiO_3_ solid solution is also indicated in this figure. Our objective is to systematically explore the complex chemical search space and identify potentially new piezoelectric materials that have high *T*_C_. In this article, we report our computational work, where we have focused particularly on identifying a suitable Me^3+^ cation (which is weakly ferroelectrically active and occupies the octahedral site of the perovskite lattice) that can significantly enhance the *T*_C_ of BiMeO_3_–PbTiO_3_ solid solution. The distinction between strong and weak ferroelectric activity was made based on the degree of off-centring tendency of Me cations in MeO_6_ octahedra. Filled circles, Me cations that show strong ferroelectric activity; filled squares, Me cations that show weak ferroelectric activity; filled triangles, Me cations that show strong and weak ferroelectric activity. (Online version in colour.)

The chemical search space of known and predicted perovskite-based ferroelectric compounds in BiMeO_3_–PbTiO_3_ solid solution is mapped in figure 1, where Me is a single cation with charge 3+ or a combination of two different cations (Me_1/2_Me_1/2_, Me_2/3_Me_1/3_ and Me_3/4_Me_1/4_) with an average charge 3+, occupying the octahedral site of the perovskite lattice (Eitel *et al*. 2001; Grinberg *et al*. 2005; Suchomel & Davies 2005; Stein *et al*. 2006; Grinberg & Rappe 2007). The solid solutions were classified based on the chemical origin of ferroelectric instability caused by Me cations. The distinction between strong (filled red circles) and weak (filled green squares) ferroelectric activity was made based on the degree of off-centring tendency of Me cations in MeO_6_ octahedra. Clearly, the search space is sparse in the high-temperature region, and our goal is to explore the vast combinatorial search space and identify new high-temperature piezoelectric chemistries. In this work, we have focused primarily on identifying a new Me^3+^ cation that satisfies the following conditions:

— it must show weak ferroelectric activity;
— BiMeO_3_ must have a stable perovskite structure at ambient or non-ambient (high-pressure/-temperature) conditions; and
— the resulting BiMeO_3_–PbTiO_3_ solid solution should have a high *T*_C_.

We explore a data-driven methodology that involves applying statistical learning tools to analyse correlations between numerous scalar descriptors of electronic and crystal structure parameters of known perovskite piezoelectric compounds and using that information in turn to develop predictive models that can suggest new structure/chemistries and/or properties based purely on the formalism of statistical learning methods. This methodology is quite different from the approach that is widely reported by many groups where large numbers of high-throughput electronic structure computations are conducted to seek compound chemistries with energy minima (where data mining-related techniques are embedded in the computation to help the efficiency of the calculations); and then potentially new stable compounds are identified by identifying those that have energy minima but not reported in known experimental databases (Jóhannesson *et al.* 2002; Curtarolo *et al.* 2003; Woodley *et al*. 2004; Dudiy & Zunger 2006; Fischer *et al.* 2006; Sluiter 2007; Mohn & Kob 2009; Oganov & Valle 2009).

Our approach requires the need to carefully establish a dataset of descriptors on which we directly apply statistical learning tools. The number of parameters needed to predict even relatively simple structures can be large if one has to capture both geometrical and bonding characteristics of that crystal chemistry. One of the arguments we are trying to put forward in this paper is that although the potential number of variables can in fact be large, data dimensionality reduction and information theoretic techniques can help reduce it to a manageable number. This paper describes a data mining strategy from which effective classification and predictive models can be developed using high-dimensional information.

**Figure 2.**
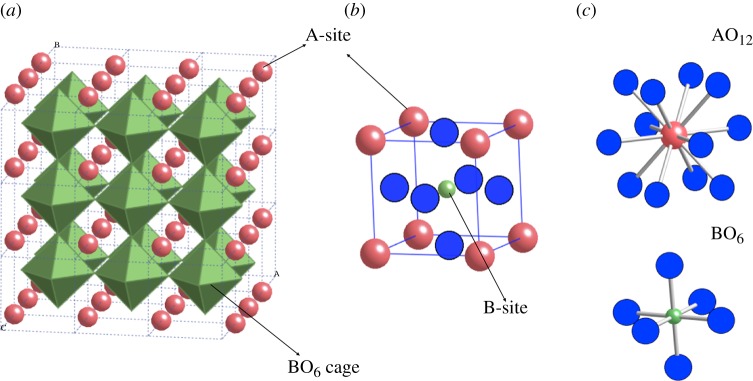
(*a*) A network of corner-sharing BO_6_ octahedra with a large A-site cation occupying the interstitial position is shown. (*b*) The simplified unit-cell representation of cubic perovskite without showing coordination. (*c*) The geometry of the building units, AO_12_ cuboctahedra and BO_6_ octahedra, with 12-coordinated A-site and 6-coordinated B-site, respectively. The description of the crystal structure in the form of structural building units presents a number of diverse choices to develop new descriptors based on the site chemistry and coordination. (Online version in colour.)

### (b) Defining the chemical search space

The search for new high-temperature piezoelectric materials by chemical modification of PbTiO_3_ perovskite at both Pb and Ti sites has been an area of considerable interest in the last decade (Sághi-Szabó *et al*. 1998; Eitel *et al*. 2001). While there are many crystal structures that may be suitable for high-temperature piezoelectric application, such as perovskites, langasites (Damjanovic 1998) and perovskite-like layered structures (Yan *et al*. 2009), we are interested in perovskites because they have the best combination of high temperature and piezoelectric properties compared with other structures, and many perovskites are also ferroelectrics, which can be used as piezoelectric materials when poled (Cohen 2008; Rödel *et al*. 2009). The crystal structure of an ideal perovskite crystal is shown in figure 2. Following the discovery of the crucial role of Bi in enhancing the ferroelectric properties in PbTiO_3_ (Íñiguez *et al*. 2003), numerous experimental and theoretical studies focusing on BiMeO_3_–PbTiO_3_ solid solutions were carried out (where Me represents a single cation with charge 3+ or a combination of cations with an average charge 3+) with the further objective of identifying a potential Me cation that can maximize both Curie temperature and ferroelectric properties of the solid solution (Suchomel & Davies 2004, 2005; Grinberg *et al*. 2005; Stein *et al*. 2006; Stringer *et al*. 2006; Chen *et al*. 2007, 2009; Grinberg & Rappe 2007). The key findings from the earlier studies are summarized below:

— Enhancement of ferroelectric properties and Curie temperature owing to the presence of strongly ferroelectrically active Me cations (e.g. Ti^4+^, Zn^2+^, Fe^3+^, etc.). These strongly ferroelectrically active Me cations cause hybridization of Me–O bonds in MeO_6_ octahedra, leading to distortions resulting in significant ionic displacement from the ideal position (Cohen 1992, 2008; Rödel *et al*. 2009). The ionic displacements were responsible for enhanced polarization and ferroelectric properties. Some examples of compounds with strongly ferroelectrically active Me cations are BiFeO_3_–PbTiO_3_ and Bi(ZnTi)O_3_–PbTiO_3_.
— On the other hand, it was found that the presence of weakly ferroelectrically active Me cations (e.g. Sc^3+^, Mg^2+^ and Yb^3+^) can also enhance the high-temperature ferroelectric properties. In this case, the Me cations do not lead to hybridization of Me–O bonds, whereas the steric effect causes the Pb/Bi cation to avoid the larger Me/Ti cation owing to the larger wave-function overlap (therefore stronger Pauli repulsion) and move towards the smaller cation. The stronger repulsion leads to increased Pb/Bi cation displacement, which in turn results in enhanced ferroelectric behaviour (Grinberg *et al*. 2005). Some examples of compounds with weakly ferroelectrically active Me cations are BiScO_3_–PbTiO_3_ and BiYbO_3_–PbTiO_3_.

Our chemical search space is defined in electronic supplementary material, figure S1, and we have focused particularly on identifying a suitable BiMeO_3_ perovskite end member, where Me is a single cation that is weakly ferroelectrically active with a formal charge 3+ and that can form a solid solution with PbTiO_3_ at ambient conditions.

## 3. Statistical learning computational strategy

### (a) Introduction to tolerance factor–T_*C*_ model

**Figure 3.**
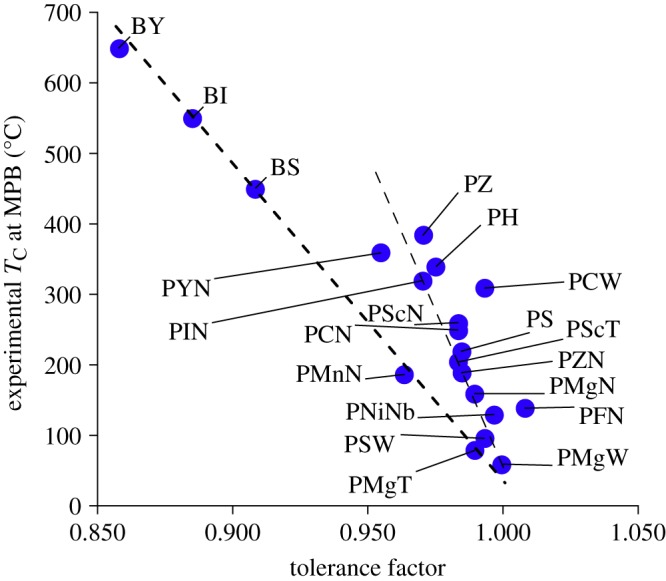
The univariate tolerance factor–*T*_C_ model of Eitel *et al*. (2001) is shown here. The shortcomings of the univariate tolerance factor–*T*_C_ model are clearly noticeable as the data show significant scatter owing to the presence of two clusters of compounds with different physics. This indicates that the tolerance factor is only a necessary condition and not sufficient for modelling *T*_C_. We have addressed the shortcomings of the tolerance factor–*T*_C_ model by developing a multivariate model that considers six key crystal chemical descriptors instead of only the tolerance factor. Notation for chemical compounds and parameters are described in the electronic supplementary material. (Online version in colour.)

 Eitel *et al*. (2001) first discovered the existence of an apparent linear relationship between tolerance factor of ABO_3_ end-member compositions and Curie temperature at MPB for a large number of ABO_3_–PbTiO_3_ solid solutions, although there was some significant scatter (figure 3). Grinberg *et al*. (2005) later addressed this scatter by identifying that the data fall into two clusters, and they showed that both clusters exhibited a linear dependence of Curie temperature on the end-member tolerance factor but had different slopes. The physical reasons behind the two slopes were correlated to the differences in the ferroelectric activity of various B-site cations of the ABO_3_ end-member compositions. While both models can be applied to quantitatively predict the *T*_C_, neither predicts the perovskite phase stability of the ABO_3_–PbTiO_3_ solid solution. This is a major shortcoming because only those ABO_3_–PbTiO_3_ solid solutions that form a pure perovskite phase at ambient conditions are technologically useful (Grinberg *et al*. 2005).

We have collectively addressed the above-mentioned shortcomings of the tolerance factor–*T*_C_ model in a couple of ways. Firstly, by considering additional crystal chemical descriptors, a reasonably accurate multivariate model was developed (described in §4*b*) using linear manifold methods for quantitatively predicting the *T*_C_ at MPB of ABO_3_–PbTiO_3_ solid solutions. To reduce the scatter, instead of including all ferroelectric ABO_3_–PbTiO_3_ chemistries that contain both strongly and weakly ferroelectrically active cations, we have typically considered end members that belong to Pb(B_1_B_2_)O_3_ and BiMeO_3_ perovskites, where B_1_, B_2_ and Me are cations that occupy the octahedral site of the perovskite lattice and Me cation is weakly ferroelectrically active. By clearly defining our chemical search space in this manner, we focus on the relevant physics that best describes our objective.

Secondly, in order to determine the perovskite phase stability of the ABO_3_–PbTiO_3_ solid solution, we have developed an independent classification model based on information theory concepts (e.g. Shannon entropy) that tracks which combination of parameters influences the perovskite structural stability by partitioning a high-dimensional dataset. As noted by Karnani *et al*. (2009), natural data structures, such as genomes, books, file systems and data servers, are repositories of information that share common characteristics. Also, they display skewed distributions and hierarchical organization, which certainly applies to crystallographic data. The physical representation of information allows us to understand that these ubiquitous characteristics are consequences of the second law. Thus, by combining the linear manifold methods with the information theory concepts, we can identify new high-temperature piezoelectric materials.

### (b) Informatics-based computational strategy

Our computational logic for designing new high-temperature piezoelectric chemistries is summarized in the form of a flow chart in the electronic supplementary material, figure S2. The logic involves three steps. (i) Identification of a relevant descriptor set that fully describes the high-temperature behaviour of ABO_3_ perovskites. Thirty attributes were screened using principal component analysis (PCA) and a reduced set of six key attributes was identified that showed high correlation with the transition temperature. (ii) Development of a robust multivariate model using partial least squares (PLS) that predicts *T*_C_ at MPB of ABO_3_–PbTiO_3_ solid solutions. By applying the PLS model, new candidate chemistries were identified that are suitable for high-temperature applications. (iii) Screening for the piezoelectric behaviour in the new candidate chemistries by testing the perovskite structural stability of ABO_3_ end members. For this purpose, new classification models were developed using a recursive partitioning strategy. The outcome of this analysis is important for determining whether it is possible to synthesize a pure perovskite phase in the ABO_3_–PbTiO_3_ solid solution. Only those ABO_3_ end members that were classified to have a stable perovskite structure-type by recursive partitioning were chosen and identified as potential high-temperature piezoelectric materials. The mathematics of PCA, PLS and recursive partitioning in the context of our specific datasets is summarized in the electronic supplementary material.

Before elaborating on the data mining methods, we need to address the obvious concern that at first glance the statistical learning methods do not in themselves explicitly solve the energy minimization problem that the physics-based calculations do. However, this concern is addressed collectively in a couple of ways. The first is that we are searching for a high-dimensional correlation between attributes of compounds that already exist and hence are by definition stable. In fact, a corollary to this point is that mathematically we are using convex optimization methods that help to ensure we have a global minimum (Izenman 2008). Second, we test the validity of our models with respect to the target materials properties (i.e. Curie temperature in this case) by using well-established and robust methods for being able to reproduce the known data, to give us the statistical confidence of the models we develop.

**Table 1.** Enumeration of 30 descriptors used in the principal component analysis (PCA) for identifying the relevant inorganic gene is given in this table. The underlying rationale behind choosing these different attributes associated with crystal geometry, bonding, thermodynamics and electronic structure was to fully describe the crystal chemistry of perovskite-based compounds that is relevant for modelling the ferroelectric behaviour, and the search was motivated by the past experimental and theoretical work of Abrahams *et al*. (1968), Igarashi *et al*. (1987), Singh *et al*. (1988), Ravez *et al*. (1997), Goudochnikov & Bell (2007) and Grinberg & Rappe (2007).

abbreviation	description
*r*_A_(Å)	Shannon’s (1976) ionic radii of A-site (12-coordination)
*r*_B_(Å)	Shannon’s ionic radii of B-site (6-coordination)
*t*	tolerance factor calculated using ionic radii
*d*_A–O_(Å)	ideal A–O bond distance (Brese & O’Keeffe 1991)
*d*_B–O_(Å)	ideal B–O bond distance
*t*_BV_	tolerance factor calculated using *d*_A–O_ and *d*_B–O_
*A*_EA_(kJ mol^−1^)	A-site electron affinity (Hotop & Lineberger 1985)
*A*_EFF–S_	A-site effective nuclear charge—Slater scale (Slater 1930)
*A*_EFF–C_	A-site effective nuclear charge—Clementi scale (Clementi & Raimondi 1963)
*A*_EFF–F_	A-site effective nuclear charge—Froese-Fisher scale (Froese-Fischer 1972)
*B*_EFF–S_	B-site effective nuclear charge—Slater scale
*B*_EFF–C_	B-site effective nuclear charge—Clementi scale
*B*_EFF–F_	B-site effective nuclear charge—Froese-Fisher scale
*A*_WS_(Å)	A-site Wigner–Seitz cell radius (Skriver 2004)
*B*_WS_(Å)	B-site Wigner–Seitz cell radius
*A*_EN–P_	A-site electronegativity—Pauling scale (Pauling 1960)
*A*_EN–AR_	A-site electronegativity—Allred–Rochow scale (Allred & Rochow 1958)
*A*_EN_(eV)	A-site electronegativity—absolute scale (Pearson 1988)
*B*_EN–P_	B-site electronegativity—Pauling scale
*B*_EN–AR_	B-site electronegativity—Allred–Rochow scale
*B*_EN_(eV)	B-site electronegativity—absolute scale
*D*_A_(Å)	ionic displacement (Grinberg & Rappe 2007) of A-site
*D*_B_(Å)	ionic displacement of B-site
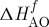 (J mol^−1^)	enthalpy of formation (Saxena 1993) of A oxide
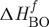 (J mol^−1^)	enthalpy of formation of B oxide
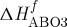 (J mol^−1^)	enthalpy of formation of ABO_3_
*a*(Å)	lattice constant (Matsui & Nomura 1981)
*b*(Å)	lattice constant
*c*(Å)	lattice constant
*V* /*Z*(Å^3^)	volume of unit cell/coordination number
*T*_t_(K)	transition temperature

## 4. Results and discussion

### (a) Identifying the relevant descriptor set: the inorganic genes

As noted above, the tolerance factor as the sole figure of merit to design new high-temperature piezoelectric perovskite compounds appears to be insufficient. To look beyond the tolerance factor to predict new high-temperature piezoelectric materials, we have surveyed over 30 different attributes (table 1) associated with crystal geometry, bonding, thermodynamics and electronic structure of 22 simple ABO_3_ perovskite chemistries with known transition temperatures (Shannon 1976; Matsui & Nomura 1981; Saxena 1993; Emsley 1998; Brown 2002; Suh & Rajan 2005; Goudochnikov & Bell 2007; Grinberg & Rappe 2007; Makov *et al*. 2009; Pettersson *et al*. 2009; Rajan 2010). The transition temperature of an ABO_3_ compound is defined as the temperature when the crystal structure of ABO_3_ changes from low symmetry to the highest possible symmetry. While not all of the ABO_3_ compounds assessed are ferroelectric, the objective of this work is unaffected, since the final goal is to suggest new perovskite-based end members forming solid solutions with PT. Alloying an ABO_3_ perovskite compound with PbTiO_3_ has the potential to lead to a high piezoelectric characteristic in the resulting ABO_3_–PbTiO_3_ ceramic (Grinberg & Rappe 2004).

To identify the complex relationships between physical properties and crystal chemistry and geometry from the existing knowledge base, PCA is employed (Ericksson *et al*. 2001; Rajan 2005; Ringnér 2008). The input *X*={*x*_1_,*x*_2_,*x*_3_,…,*x*_*n*_}∈Re^*n*×*d*^ (where *n*=22 and *d*=30 denote the number of ABO_3_ compounds and the number of physical attributes quantifying each ABO_3_ compound, respectively) is initially preprocessed by mean-centring and standardization. PCA reduces the dimensionality of the data by identifying new latent variables (called principal components, PCs) that capture the largest amount of variation in the data. Each PC is a linear combination of the weighted contribution of each attribute. By comparing the magnitude and direction of the weighted contribution from each attribute, the correlation structure in the high-dimensional data is discovered).

**Figure 4.**
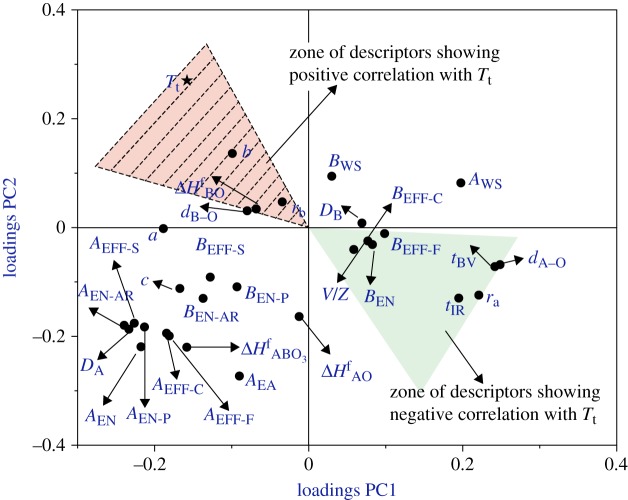
Loadings plot between PC1 and PC2 showing the interactions of 30 descriptors captured by PCA. Based on the angle *θ*, the degree of correlation between the target variable and other attributes is established. Two zones are marked in the figure that show a strong correlation with the target variable (*T*_t_): the red zone (with stripes) signifies attributes that show positive correlation with *T*_t_ and the green zone (no stripes) signifies variables that show negative correlation with *T*_t_. The abbreviations of the attributes are provided in table 1. (Online version in colour.)

 Figure 4 (referred to as a loading plot) shows the uncovered correlations between the physical attributes for the first two PCs. The transition temperature (*T*_t_) is the target variable against which all correlations are computed. As we are using linear manifold methods, we have employed Euclidean geometrical mapping to help interpret these plots. The degree of correlation between any attribute and *T*_t_ is determined by the cosine of the angle (*θ*) between the attribute and *T*_t_ (angle between attribute origin–*T*_t_) within the loading plot. If *θ*=0^°^, the attribute and *T*_t_ are highly positively correlated, if *θ*=180^°^, then they are highly negatively correlated and if *θ*=90^°^, there is no correlation between the attribute and *T*_t_. In figure 4, two zones that show the strongest correlation of the attributes with *T*_t_ are explicitly marked, with the assumption that the first two PCs capture such a high percentage of the data’s information that the other PCs do not need to be explicitly considered. The attributes *r*_*B*_ (ionic radii of B-site), *d*_B–O_ (ideal B–O bond distance based on the bond-valence model), Δ*H*_fBO_ (enthalpy of formation of BO oxide) and b (lattice constant) correlate positively with *T*_t_, while *r*_*A*_ (ionic radii of A-site), *d*_A–O_ (ideal A–O bond distance based on the bond-valence model), *t* (tolerance factor calculated using ionic radii), *t*_BV_ (tolerance factor calculated using the bond-valence method), *B*_EN_ (B-site electronegativity—absolute scale), *B*_Eff_ (B-site effective nuclear charge) and *V*/*Z* (volume of unit cell/coordination number) correlate negatively with *T*_t_. Our PCA model reproduces the well-known inverse linear relationship between tolerance factor (*t*) and *T*_t_. Based on the removal of redundancy and consideration of available data, we have determined that six attributes (*r*_A_, *t*, *B*_EN_, *d*_A–O_, *r*_B_ and *d*_B–O_) are appropriate for describing *T*_t_. By identifying these attributes, we can more fully describe the high-temperature behaviour than possible by only considering the tolerance factor (*t*), and the selection of only the highly correlated attributes ensures the robustness of the model.

### (b) Identifying new high-temperature perovskites: developing a ‘QSPR’

**Figure 5.**
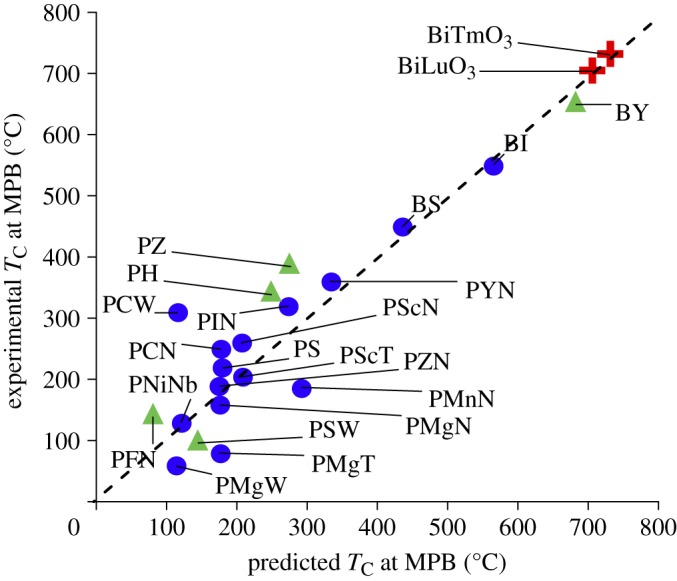
Multivariate predicted model (abscissa) in comparison with the measured *T*_C_ as reported in the literature (Eitel *et al*. 2001; Grinberg *et al*. 2005) is shown for the PbTiO_3_ end members. The model was developed by using 15 chemistries and tested for five chemistries. The new figure of merit is *T*_C_=−(789.912×*t*)−(153.932×*r*_A_)+(1013.981×*r*_B_)+(796.5864×*d*_B–O_)−(138.9× *d*_A–O_)−(55.6076×*B*_EN_)−526.537. Based on the new figure of merit, the *T*_C_ of new piezoelectric chemistries BiTmO_3_–PT and BiLuO_3_–PT were predicted to be 730^°^C and 705^°^C, respectively (labelled red in the figure). It should be noted that the *T*_C_ of BiTmO_3_–PT and BiLuO_3_–PT plotted in the figure is only the predicted value and needs to be experimentally validated. Notation for chemical compounds and parameters are described in the electronic supplementary material. Filled circles, training set; filled triangles, test set; plus symbols, new predictions. (Online version in colour.)

To test for high-*T*_C_ piezoelectric materials, we have applied PLS regression (Ericksson *et al*. 2001) to predict *T*_C_ at the MPB of the end-member PbTiO_3_ solid solution. PLS is particularly suitable for handling sparse data with strongly correlated attributes. The piezoelectric materials database for predicting *T*_C_ as a function of six attributes (*r*_A_, *t*, *B*_EN_, *d*_A–O_, *r*_B_ and *d*_B–O_) is taken from the published work of Eitel *et al.* (2001) and Grinberg *et al.* (2005). This new QSPR formulated using PLS is given by

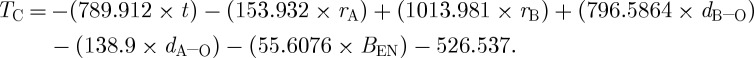
Fifteen compounds were used for training the model and an independent set of five compounds (not used during the training) was used for testing (figure 5). Our QSPR model takes into account the physics of mismatch of bond lengths (*t*), ionic size (*r*_A_ and *r*_B_), bond lengths (*d*_A–O_ and *d*_B–O_) and chemical bonding at the B-site (*B*_EN_), thereby accounting for a far greater diversity of attributes in comparison to the previous model where only mismatch of bond lengths was considered. Some of the descriptors captured in our QSPR model are also in the original description of the tolerance factor. However, only two (*r*_B_ and *r*_A_) of the six descriptors are explicitly used in the tolerance factor formulation,

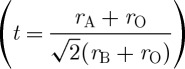
while the other four descriptors are not explicitly used. For end members that had more than one cation in the octahedral site, such as Pb(B_1_B_2_)O_3_, we considered the arithmetic mean value of B_1_ and B_2_. It should be noted, although not elaborated in this paper, that the classification of Me ions into weakly and strongly ferroelectric active species can be accomplished by exploring more descriptors such as polarizability, ionic valence and ionic size.

The additional diversity of the QSPR model has a clear advantage as compared with the model based solely on tolerance factor. For many compounds, the QSPR model is in reasonable agreement with the tolerance factor model. However, in some cases, the mismatch of bond length is not sufficient for modelling the physics of the system. For the systems predicted here, BiLuO_3_–PbTiO_3_ is predicted to have a higher *T*_C_ than any systems included in the training dataset; however, this result is not found when using the tolerance factor model. Therefore, we conclude that our developed QSPR is highly robust in predicting the *T*_C_ of unknown compounds (figure 5) and has a more broad significance when applied to new materials. Based on this QSPR model, a search of all the elements in the periodic table that best satisfy the correlation criterion involving the combination of attributes was performed. The search has resulted in generating four new ABO_3_ chemistries (BiTmO_3_, BiLuO_3_, BiHoO_3_ and BiErO_3_) as potential high-*T*_C_ materials. Having identified the new chemistries, we then tested them for their crystal structure-type.

### (c) Screening for piezoelectric behaviour: ‘sequencing the gene’

To test for the perovskite structural stability, a new classification model was developed using a recursive partitioning strategy (Witten & Frank 2000; Hall *et al*. 2009) on a large database (taken from the work of Zhang *et al*. 2007 and references therein) of 355 ABO_3_ stoichiometric compounds (227 perovskites and 128 non-perovskites) to track which combination of parameters influences the perovskite structural stability by partitioning a high-dimensional dataset. The outcome of this analysis is important for determining whether it is feasible to synthesize a pure perovskite phase in the BiBO_3_–PbTiO_3_ solid solution (where B=Tm, Lu, Ho, Er). Our hypothesis is, if BiTmO_3_, BiLuO_3_, BiHoO_3_ and BiErO_3_ compounds are predicted to have a stable perovskite structure-type at ambient or non-ambient (high pressure/temperature) condition, then we propose that it is possible to experimentally obtain a pure perovskite phase in BiBO_3_–PbTiO_3_ solid solution (where B=Tm, Lu, Ho, Er). Here, we explain the relevance of this hypothesis using a few examples based on experimental observations.

It is well known that obtaining a pure Bi-based perovskite is difficult under conventional processing methods at ambient conditions. For example, a pure perovskite phase in BiScO_3_ is synthesized only at 6 GPa pressure and 1140^°^C temperature (Belik *et al*. 2006*a*,*b*) and in BiMnO_3_ a pure perovskite phase is obtained only at pressures greater than 4 GPa and 750^°^C temperature (Montanari *et al*. 2005). However, solid solutions of BiScO_3_–PbTiO_3_ (Zhang *et al*. 2003) and BiMnO_3_–PbTiO_3_ (Woodward & Reaney 2004) have been experimentally synthesized and are shown to have a pure perovskite phase. Even in the case of very low tolerance factor end members such as BiYbO_3_ (tolerance factor=0.857), there are experimental reports that confirm the limited solubility of BiYbO_3_ in PbTiO_3_. Feng *et al*. (2009) using conventional ceramic processing methods synthesized a solid solution of 0.05BiYbO_3_–0.95PbTiO_3_ with the highest perovskite phase purity of 97.83 per cent. Obtaining a pure perovskite phase in BiYbO_3_ when synthesized at ambient conditions is extremely difficult (Drache *et al*. 2004), and we note that there is no experimental or theoretical study on structural phase transitions in BiYbO_3_ at high-pressure/-temperature conditions. In this work, we have identified for the first time the existence of a stable perovskite structure-type in BiYbO_3_ via a recursive partitioning strategy at high-pressure/-temperature conditions, and this structural stability at high-pressure/-temperature conditions explains the limited solubility of BiYbO_3_ in PbTiO_3_ at ambient conditions. Alloying BiYbO_3_ with PbTiO_3_, which has a large *c*/*a* ratio, can help stabilize a perovskite phase by applying chemical pressure (Ahart *et al*. 2008).

In this work, we apply our classification model to qualitatively determine the feasibility of synthesizing a pure perovskite phase in the BiBO_3_–PbTiO_3_ solid solution (where B=Tm, Lu, Ho, Er). In order to capture the physics of perovskite stability at high-pressure/-temperature conditions, we have included ABO_3_ perovskite compounds such as BiScO_3_ (Belik *et al*. 2006*a*,*b*), BiMnO_3_ (Montanari *et al*. 2005), BiAlO_3_ (Belik *et al*. 2006*a*,*b*), NaSbO_3_ (Mizoguchi *et al*. 2004) and YInO_3_ (Shannon 1967) that are experimentally known to have a stable perovskite structure-type only at extreme pressure/temperature conditions. Therefore, the design rules that we extract from our classification model are applicable to identify new perovskites at both ambient and high-pressure/-temperature conditions. Using the Shannon entropy as a selection criterion, a hierarchical set of design rules was formulated to develop classification schemes that hitherto have been approached by empirical observation (Plenio & Vitelli 2001; Shell 2008; Karnani *et al*. 2009).

The expected information required to classify an ABO_3_ compound solely based on its proportion in the database *D* is given by the Shannon entropy *H*(*D*), which is defined as

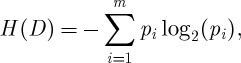
where *p*_*i*_ is the probability that an arbitrary tuple in ‘*D*’ belongs to perovskite crystal structure or not. A log function of base 2 is used, because the information is encoded in bits and *m* is an integer with distinct values defining *m* distinct classes (Han & Kamber 2006). We formulated our recursive partitioning as a binary classification problem. Further details on the construction and interpretation of the dendrogram are provided in the electronic supplementary material.

The aim of the classification is to track precisely which and how variables contribute to perovskite structural stability. The output from a recursive partitioning analysis is a dendrogram (or a tree diagram) with branches grown on each node (attribute) to classify whether a particular ABO_3_ compound forms a perovskite crystal structure. The advantage of the recursive partitioning method is that it can efficiently model nonlinear relationships in any arbitrary form even when the attributes show strong interactions (Hawkins *et al*. 1997). Our recursive partitioning model classified 336 out of 355 compounds accurately (95% accuracy), and the model was validated by a standard 10-fold cross-validation technique used in statistics.

**Figure 6.**
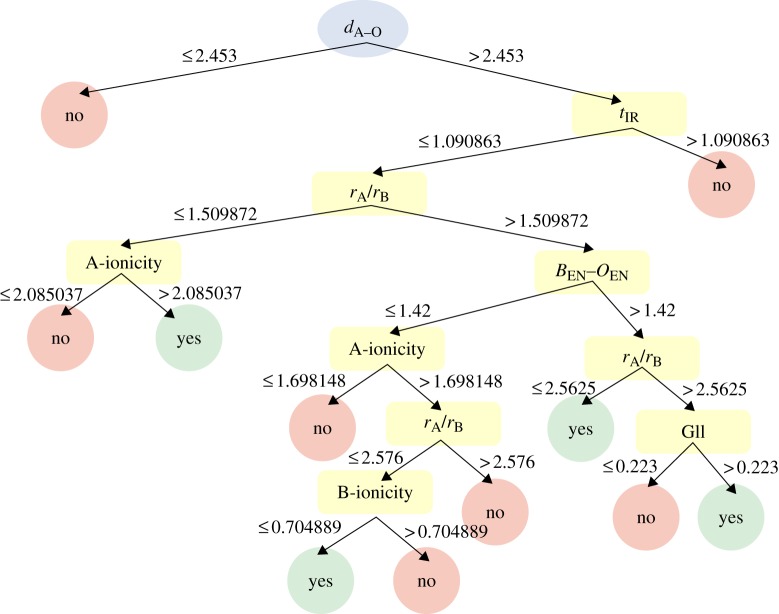
The dendrogram (or tree diagram) classification model developed based on the recursive partitioning method for identifying new potentially stable perovskite compounds is shown. We used the Shannon entropy as a selection criterion to identify key descriptors, and a hierarchical set of design rules were formulated to develop classification schemes that have been approached by empirical observation. The leaf nodes that are labelled ‘yes’ or ‘no’ indicate compounds that may have a stable perovskite structure-type or not a perovskite, respectively. From the dendrogram, 11 design rules were formulated for testing the perovskite structural stability. By applying the dendrogram to the four candidate high-temperature materials BiErO_3_, BiHoO_3_, BiTmO_3_ and BiLuO_3_, only two compounds, BiTmO_3_ and BiLuO_3_, were identified as having the stable perovskite crystal structure at high-pressure/-temperature conditions. As a result, BiTmO_3_–PbTiO_3_ and BiLuO_3_–PbTiO_3_ solid solutions were identified as new perovskite compounds with a significantly high *T*_C_ while having piezoelectric behaviour. The dendrogram application of other Bi-based systems BiMEO_3_, where ME=Cr, Co, Ga and Ni, also identifies them as having the perovskite crystal structure in agreement with the literature (Ishiwata *et al.* 2002; Baettig *et al.* 2005; Goujon *et al.* 2008; Oka *et al.* 2010). In the dendrogram, *d*_A–O_ is the ideal A–O bond length calculated based on the bond-valence method, *t*_IR_ is the tolerance factor from ionic radii data, *r*_A_ is ionic radii (Shannon’s scale) of the A-site cation with coordination number 12, *r*_B_ is the ionic radii (Shannon’s scale) of the B-site cation with coordination number 6, *B*_EN_–*O*_EN_ is the electronegativity difference (Pauling’s scale) between B-site and O-site, A-ionicity is the product of *r*_A_/*r*_O_ and *A*_EN_–*O*_EN_, B-ionicity is the product of *r*_B_/*r*_O_ and *B*_EN_–*O*_EN_ and GII is the global stability index (Zhang *et al*. 2007). (Online version in colour.)

The dendrogram model used for predicting new perovskites is shown in figure 6. According to the dendrogram, *d*_A–O_ (ideal A–O bond length calculated based on the bond-valence method) is the most significant attribute impacting the phase stability of perovskite compounds, followed by the tolerance factor. The leaf nodes that are labelled ‘yes’ and ‘no’ indicate compounds that may have a stable perovskite structure-type or not a perovskite, respectively. From the dendrogram, design rules were extracted for predicting new potentially stable perovskite compounds. Of the 227 perovskite compounds, 184 obeyed the following rule: if *d*_A–O_>2.453 and *t*_IR_≤1.090863 and *r*_A_/*r*_B_>1.509872 and *B*_EN_–*O*_EN_>1.42 and *r*_A_/*r*_B_≤2.5625, then the ABO_3_ compound is a perovskite, where *d*_A–O_ is the ideal bond length based on the bond-valence model, *t*_IR_ is the tolerance factor calculated using ionic radii, *r*_A_/*r*_B_ is the ionic radii ratio of A-site to B-site and *B*_EN_–*O*_EN_ is the electronegativity difference (Pauling scale) between B-cation and O-anion. A total of 11 design rules were formulated for testing the perovskite structural stability.

By applying the dendrogram to the four candidate ABO_3_ compounds, only two compounds, BiTmO_3_ and BiLuO_3_, were identified as having a stable perovskite crystal structure at high-pressure/-temperature conditions. Experimental synthesis of BiTmO_3_ and BiLuO_3_ compounds at ambient pressure has been attempted in the past but was unsuccessful in synthesizing a pure perovskite phase (Drache *et al*. 2005); however, there are no data available on synthesizing BiTmO_3_ and BiLuO_3_ compounds at high-pressure/-temperature conditions. Therefore, we predict for the first time the existence of a stable perovskite phase in BiTmO_3_ and BiLuO_3_ compounds at high-pressure/-temperature conditions. This result indicates that Tm^3+^ (thulium) is the largest cation (with an ionic radius of 0.88 Å in sixfold coordination) that can occupy the octahedral site of a BiMeO_3_ perovskite lattice without impacting its phase stability. The dendrogram also predicts the existence of a stable perovskite phase in BiYbO_3_ at high-pressure/-temperature conditions. BiYbO_3_–PbTiO_3_ is known as a potential high-temperature piezoelectric material (Eitel *et al*. 2001; Feng *et al*. 2009), and there are experimental reports that confirm the limited solubility of BiYbO_3_ in PbTiO_3_, thereby forming a solid solution (Feng *et al*. 2009). Thus, we conclude that it is possible to experimentally obtain a pure perovskite phase in BiLuO_3_–PbTiO_3_ and BiTmO_3_–PbTiO_3_ solid solutions. Based on the QSPR and the recursive partitioning model, two new perovskite end members were identified (BiTmO_3_–PbTiO_3_ and BiLuO_3_–PbTiO_3_) and predicted to have a high *T*_C_ of 730^°^C and 705^°^C at the MPB, respectively, while having piezoelectric behaviour.

The focus of this report has been solely on identifying new BiMeO_3_–PbTiO_3_ materials chemistries with higher Curie temperatures, where Me is a weakly ferroelectrically active cation with a formal charge 3+. We fully realize that other electronic structure parameters such as polarizability and other microstructural parameters play a critical role in defining a useful high-temperature piezoelectric material. This involves exploring a larger and more diverse chemical space that includes more than one Me cation that is strongly ferroelectrically active, which is presently being done, as well as experimental verification of our results, which will be reported in upcoming publications.

## 5. Summary

We have identified two new perovskite-based piezoelectric crystal chemistries, BiTmO_3_–PbTiO_3_ and BiLuO_3_–PbTiO_3_, with significantly higher Curie temperature using a highly efficient and robust computational strategy based on statistical learning and information theory concepts. The data mining strategy we have developed also permits us to identify key physical attributes that appear to govern the properties of a given crystal chemistry (e.g. piezoelectrics with a high Curie temperature), providing a mechanistic-based discovery process and not just a heuristic strategy. Finally, this paper helps to establish the efficacy of informatics as an approach to refine the chemical search space for materials discovery and to hence serve as a broader template for materials design in other applications.

## Supplementary Material

Identifying the “Inorganic Gene” for High Temperature Piezoelectric Perovskites through Statistical Learning
