# Supplementary material for: Identifying the ‘inorganic gene’ for high-temperature piezoelectric perovskites through statistical learning
Source: Proc Math Phys Eng Sci. 2011 Mar 2;467(2132):2271–90. doi: 10.1098/rspa.2010.0543 (PMC4042451; doi:10.1098/rspa.2010.0543)
Supplement: Identifying the “Inorganic Gene” for High Temperature Piezoelectric Perovskites through Statistical Learning [file rspa20100543supp1.doc]

**Identifying the “inorganic gene” for high temperature piezoelectric perovksites through statistical learning**

Prasanna V. Balachandran, Scott R. Broderick and Krishna Rajan*

Department of Materials Science and Engineering & Institute for Combinatorial Discovery, Iowa State University, Ames, IA 50011, USA.

*Corresponding author email address: [krajan@iastate.edu](mailto:krajan@iastate.edu)

**ELECTRONIC SUPPLEMENTARY MATERIALS**

Table S1 List of end members of PbTiO_3_ used for building partial least squares (PLS) model. All data is taken from the work of Eitel *et al.* (2001) and Grinberg *et al.* (2005).

| **Abbreviation** | **Solid solution of PbTiO_3_-based end members** | **Experimentally reported T_c_ at MPB (°C)** |
| --- | --- | --- |
| PMgW | Pb(Mg,W)O_3_ | 60 |
| PMgT | Pb(Mg,Ta)O_3_ | 80 |
| PNiNb | Pb(Ni,Nb)O_3_ | 130 |
| PFN | Pb(Fe,Nb)O_3_ | 140 |
| PMgN | Pb(Mg,Nb)O_3_ | 160 |
| PMnN | Pb(Mn,Nb)O_3_ | 187 |
| PZN | Pb(Zn,Nb)O_3_ | 190 |
| PScT | Pb(Sc,Ta)O_3_ | 205 |
| PCW | Pb(Co,W)O_3_ | 310 |
| PIN | Pb(In,Nb)O_3_ | 320 |
| PH | PbHfO_3_ | 340 |
| PYN | Pb(Yb,Nb)O_3_ | 360 |
| PZ | PbZrO_3_ | 385 |
| BS | BiScO_3_ | 450 |
| BI | BiInO_3_ | 550 |
| BY | BiYbO_3_ | 650 |
| PS | PbSnO_3_ | 220 |
| PCN | Pb(Co,Nb)O_3_ | 250 |
| PScN | Pb(Sc,Nb)O_3_ | 260 |
| PScW | Pb(Sc,W)O_3_ | 97 |

**
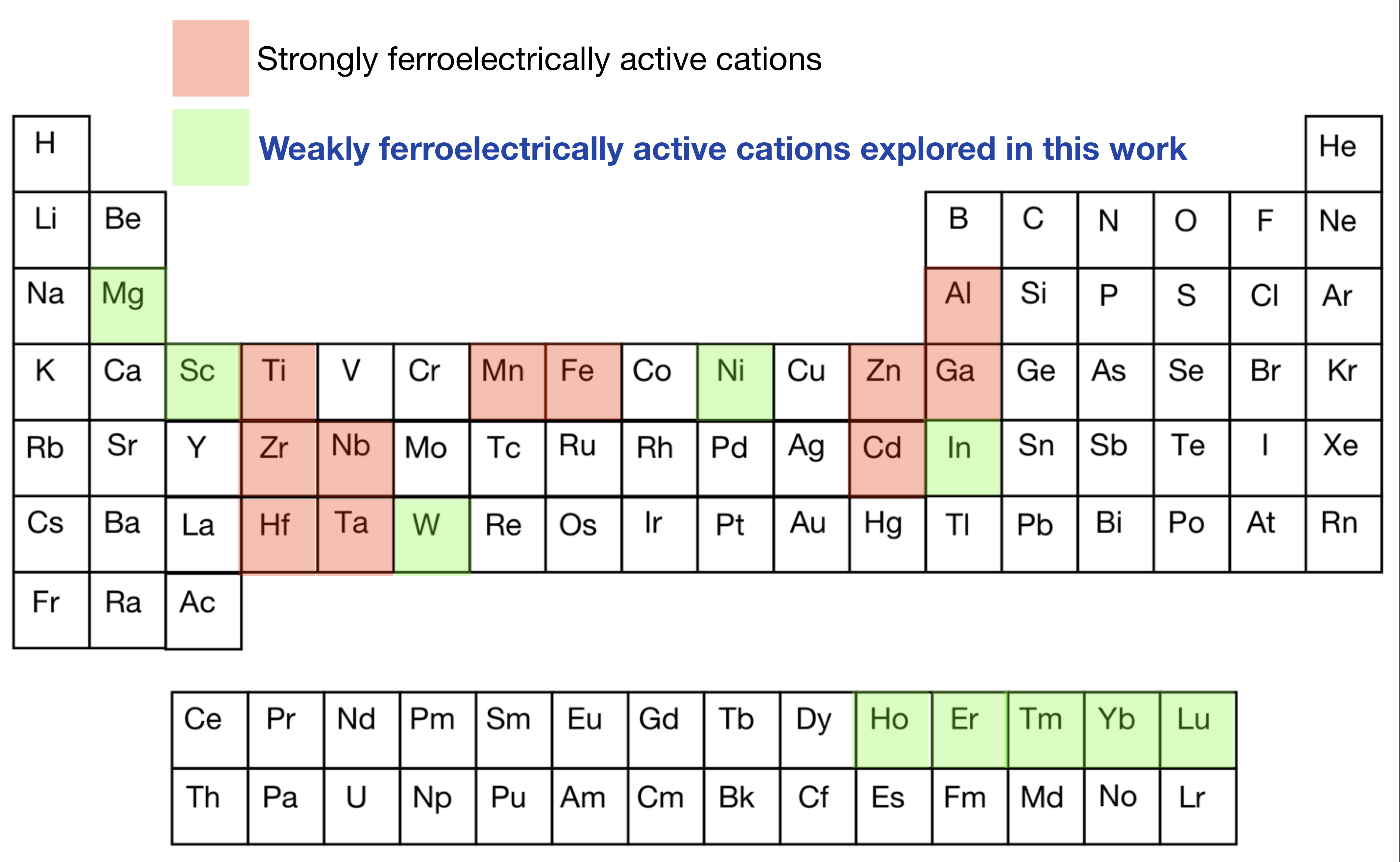
**

Figure S1. Potential octahedral site cations along with their ferroelectric activity are highlighted in this figure. Depending on the strong covalent character of Me-O bond in MeO_6_ octahedra of perovskites, Me-cations undergo ionic displacement from their ideal position. The magnitude of ionic displacement determines whether it is a strongly or weakly ferroelectrically active cation. In this work, we have focused mainly on weakly ferroelectrically active octahedral site cations (filled green squares). We are particularly interested in testing the feasibility of Me^3+^=Ho^3+^, Er^3+^, Tm^3+^ and Lu^3+^ cations to occupy the octahedral site of perovskite lattice without impacting the phase stability of BiMeO_3_-PbTiO_3_ solid solution.

**
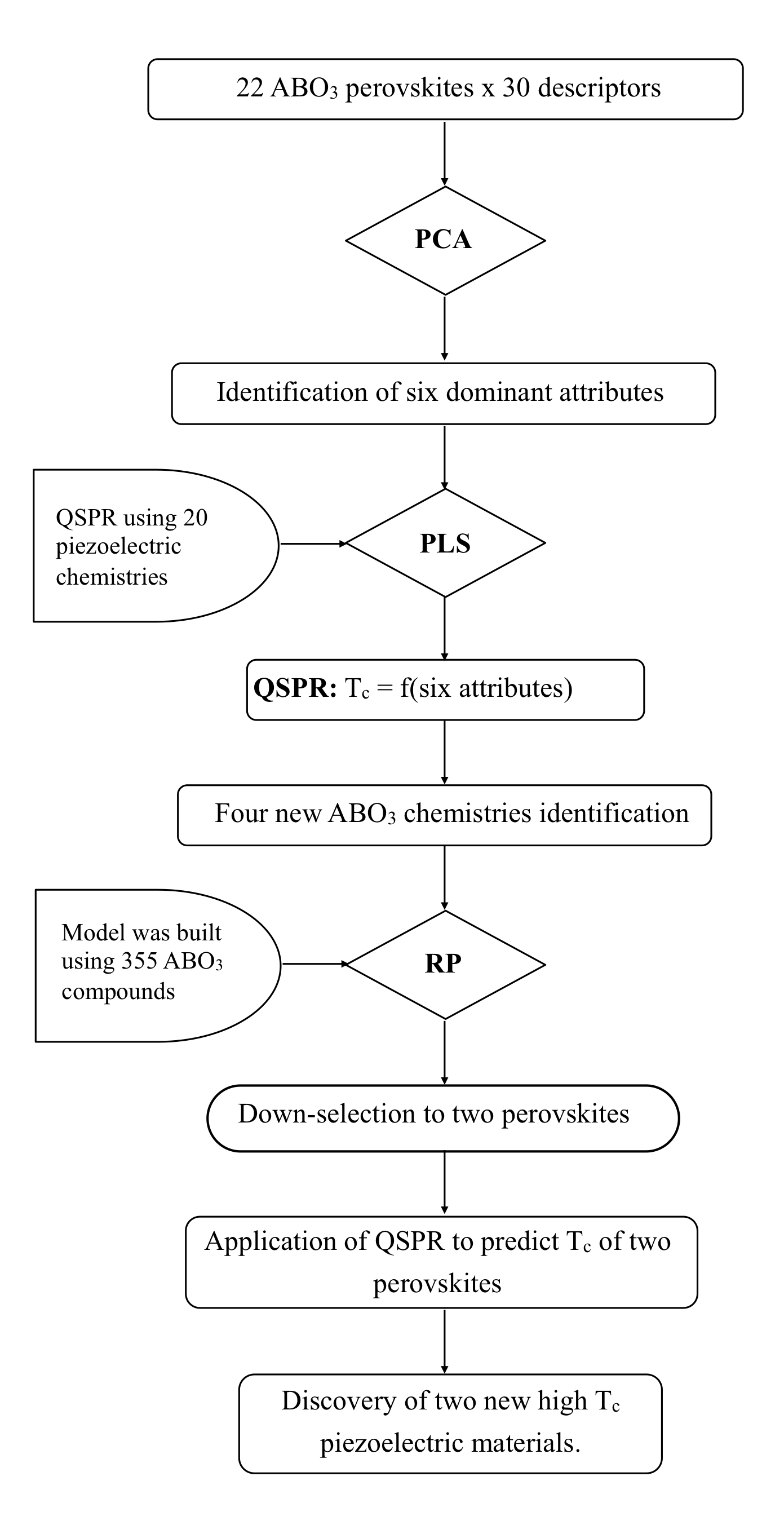
**

Figure S2. Our step-by-step computational strategy involving a hybrid combination of various statistical learning techniques is shown in the form of a flow chart. Key: T_c_ – Curie temperature of piezoelectric compounds, PCA – Principal Component Analysis, PLS – Partial Least Squares, RP – Recursive Partitioning method.

**Mathematical background**

*(a) Principal Component Analysis (PCA)*

PCA is a linear data-dimensionality reduction technique that relies on the fact that most of the descriptors are interrelated and these correlations in some instances are high. The mathematical description of PCA can be defined within the framework of an eigenvalue problem. The data manipulation involves decomposition of the input matrix (where = 22 and = 30 denote the number of ABO_3_ compounds and the number of physical attributes quantifying each ABO_3_ compound respectively) into two matrices that are orthogonal to one another.

(A1)

The matrix is called the scores matrix, is called the loadings matrix,is called the residual matrix and the subscript is the transpose of matrix operation. The loadings matrix (Fig. 2) can be understood as the weights for each original variable when calculating the principal component (PC). The matrix contains the original data in the rotated coordinate system. The mathematical analysis involves finding these “new” data matrices . The following steps describe the detailed mechanics of computing the principal components:

- Data pre-processing (optional step), which involves mean-centering and standardization. This process will eliminate any inherent bias in the dataset and after pre-processing each descriptor will have zero mean and unit variance.
- Calculating the covariance of the pre-processed data.
- Calculating the Eigen value and Eigen vectors of the covariance matrix. The Eigen vectors constitute the PCs. The corresponding Eigen values describe the amount of variance contained in the individual PCs. The first PC accounts for the maximum variance (maximum Eigen value) in the original dataset and the second PC is orthogonal to the first and accounts for the remaining variance. Thus the PC is orthogonal to all PCs and captures the largest variance in the original dataset . Normally, only those PCs are retained that contains the Eigen value greater than 1.

Thus using PCA one can compress the high dimensional data in into two or three dimensions of thereby capturing physically relevant correlations that are needed for developing quantitative structure-property relationships (QSPR).

*(b) Partial Least Squares (PLS)*

The goal of PLS is to develop a model that expresses an explicit quantitative relationship between the target property of interest (Curie temperature) and a set of correlated exploratory variables. Ifis a matrix containing the explanatory variables and is a matrix containing the target property (where is the number of piezoelectric compounds considered in the analysis), then in PLS two linear combinations of and are generated and the maximum covariance between and is calculated.

(A2)

(A3)

where and are the scores and loadings of respectively, and are the scores and loadings of respectively, andare the residuals of and respectively. Since the explanatory variables in are good predictors of
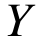
, then

(A4)

where matrix represents the difference between predicted and observed Curie temperature values.

Since
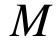
 is the scores matrix of
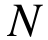
, where is the matrix that contains the weights (Eigen vectors). Now substitutingin (A4), we get

(A5)

Substitutingin (A5) implies,

(A6)

Thus the relationship between Curie temperature (
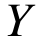
) and the correlated exploratory variables (
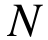
) is established using PLS. Matrix contains the regression coefficients.

*(c) Construction and Interpretation of dendrogram via Recursive partitioning*

Recursive-partitioning is an exploratory data analysis technique where we construct models by successively splitting a large dataset into increasingly homogeneous subsets until it is not feasible to continue based on some well-defined constraints. The final model from recursive partitioning is a dendrogram (also called the decision tree). Dendrogram classifies a data item by posing a series of questions about the features associated with the items. Each question is contained in a node and every internal node points to one child node for each possible answer to the question. The questions form the hierarchy, encoded as a dendrogram (Kingsford, C & Salzberg, S. L. (2008) *Nat. Biotechnol.* **26**, 1011.)

*Construction of a dendrogram*

Step 1: Development of a dataset (S) of experimentally known ABO_3_ compounds that form perovskite structure and that do not form perovskite structure.

Step 2: Append the dataset developed in step 1 with a large array of discrete crystal and electronic structure attributes for each chemical composition. Let us call this as A_k_ having *ν*-distinct values {a_1_, a_2_, …, a_ν_}.

Step 3: We begin to partition the data on some attribute, say A_k_. The goal here is to choose the best attribute that reduces the uncertainty in classifying perovskites from non-perovskites. We use the metric known as *Information gain* for this purpose. Information gain is an attribute selection measure. The attribute with the highest information gain minimizes the information needed to classify the data and reflects the least randomness in the partition. Information gain is defined as the difference between the original information requirement (H(S)) and new requirement obtained after partitioning on A_k_. In this step, we use the Shannon’s entropy metric (H(S)) to quantify the expected information.

(A8)

(A7)

In the above equation, the term $\frac{\left|S_{j}\right|}{\left|S\right|}$ acts as the weight of the *jth* partition and *m*=2 (perovskite and non-perovskite).

Step 4: Normally, dendrogram construction will be terminated with the computation of information gain. However, one drawback of information gain metric is it is biased towards the attribute that has a large number of values. To overcome the bias, the *Gain ratio* is calculated that normalizes the information gain.

(A9)

Step 5: The process is repeated for all the *k*-attributes in the set {A_k_}. The particular attribute with the highest gain ratio is chosen as the first splitting attribute.

Step 6: The above 5 steps are iteratively repeated until we have no data left to further classify according to some well-defined constraints. The above process results in the construction of a dendrogram (as shown below).

*
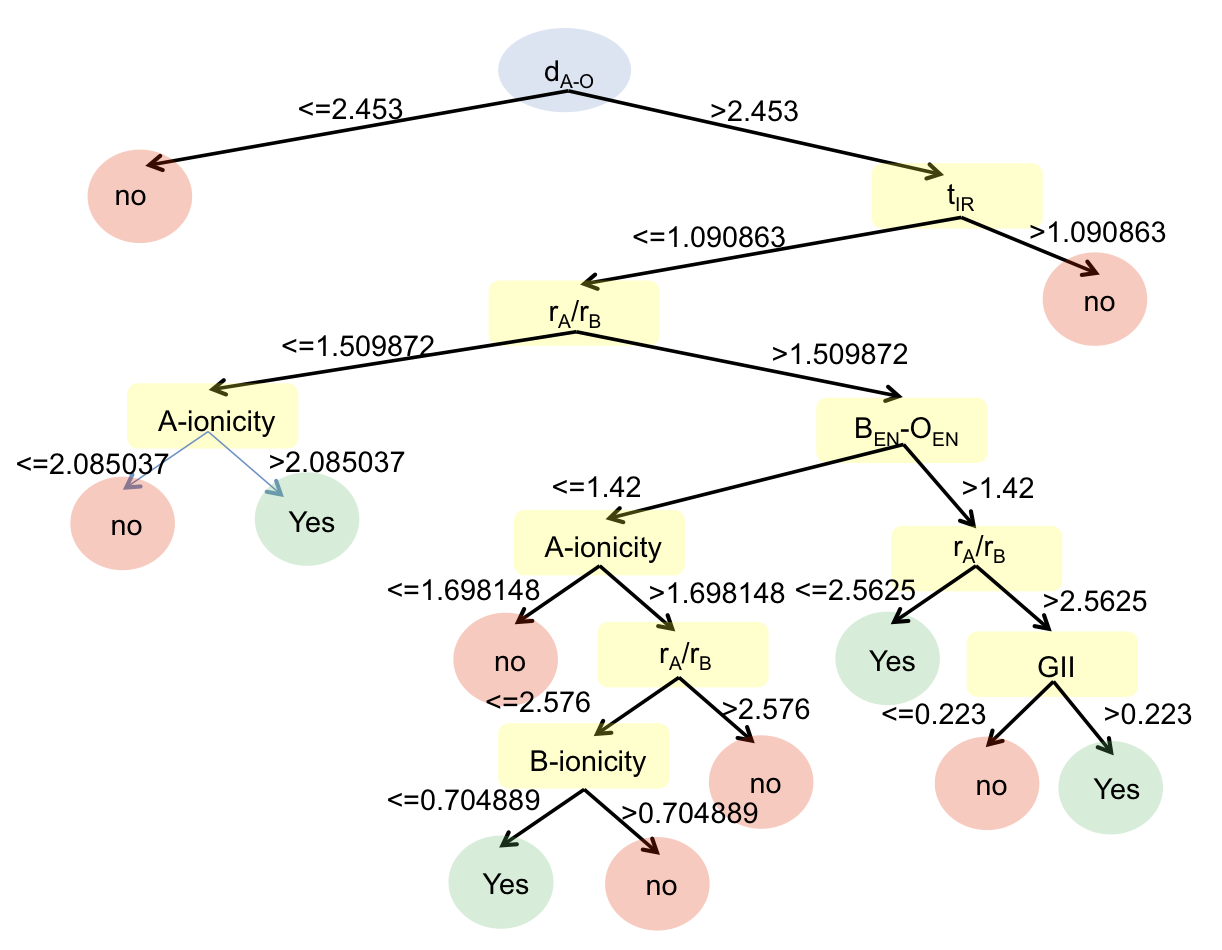
*

*Interpretation of a dendrogram*

- Each predictor variable is contained in a node and every internal node points to one terminal child node. Associated with the child node is the class assignment, which indicates whether a given chemical composition will have perovskite crystal structure or not.
- The dendrogram starts with a root node (d_A-O_) traces a path through the tree until it reaches a terminal child node (*Yes* / *No*).
- We can extract IF… THEN rules for each path from the root to leaf node that holds the structure classification.

*Examples to demonstrate how to read a dendrogram*

**Goal**: To classify the crystal structure of BiMEO_3_, ME=Cr, Ga, Co and Ni.

| Compound | d_A-O_ | Tolerance factor (t_IR_) | B_EN_-O_EN_ | r_A_/r_B_ |
| --- | --- | --- | --- | --- |
| BiCrO_3_ | 2.607 | 0.9681 | 1.78 | 2.17886 |
| BiGaO_3_ | 2.607 | 0.9706 | 1.63 | 2.16129 |
| BiCoO_3_ | 2.607 | 0.9756 | 1.56 | 2.1967 |
| BiNiO_3_ | 2.607 | 0.9756 | 1.53 | 2.2333 |

Step 1: The root node in the dendrogram is d_A-O_ and the splitting criterion is <=2.453 or >2.453. In our table, all BiMEO3 compounds have d_A-O_ = 2.607. So, we should follow the path d_A-O_ > 2.453 in the dendrogram.

Step 2: When d_A-O_ > 2.453 then the next criterion is t_IR_ (tolerance factor) and the splitting condition is <=1.090863 and >1.090863. In our case, all four BiMEO_3_ compounds have t_IR_ <=1.090863. Let us follow that path.

Step 3: When t_IR_ <= 1.090863, then the next criterion is r_A_/r_B_ and the splitting condition is <=1.509872 and >1.509872. In our BiMEO_3_ examples, all compounds have r_A_/r_B_ > 1.509872. We have to follow that path.

Step 4: When r_A_/r_B_ > 1.509872, then the next criterion is B_EN_-O_EN_ and the splitting condition is <=1.42 and >1.42. All our BiMEO_3_ compounds have B_EN_-O_EN_ > 1.42. We follow that path.

Step 5: When B_EN_-O_EN_ >1.42, then the next criterion is r_A_/r_B_ and the splitting condition is <=2.5625 and >2.5625. All our BiMEO_3_ compositions have r_A_/r_B_ <=2.5625. When we follow that path, we approach the terminal node – YES. This indicates all the BiMEO_3_ compounds have the likelihood to form perovskite structure.

Thus one can interpret the dendrogram for identifying potentially new perovskite chemistries.
